# Cost-effectiveness analysis of first-line serplulimab combined with chemotherapy for extensive-stage small cell lung cancer

**DOI:** 10.3389/fpubh.2023.1156427

**Published:** 2023-08-31

**Authors:** Xueyan Liang, Xiaoyu Chen, Huijuan Li, Yan Li

**Affiliations:** ^1^Phase 1 Clinical Trial Laboratory, Guangxi Academy of Medical Sciences and the People’s Hospital of Guangxi Zhuang Autonomous Region, Nanning, Guangxi, China; ^2^Department of Pharmacy, Guangxi Academy of Medical Sciences and the People’s Hospital of Guangxi Zhuang Autonomous Region, Nanning, Guangxi, China

**Keywords:** serplulimab, cost-effectiveness, extensive-stage small cell lung cancer, partitioned survival model, etoposide, carboplatin

## Abstract

**Background:**

For patients with extensive-stage small cell lung cancer (ES-SCLC), serplulimab plus chemotherapy is beneficial as the first-line treatment. It is uncertain whether serplulimab plus chemotherapy will be more cost-effective. The aim of this study was to evaluate from the perspective of the Chinese healthcare system to assess the cost-effectiveness of serplulimab plus chemotherapy for patients with ES-SCLC.

**Materials and methods:**

This study employed a partitioned survival model. Patients in the model were selected from ASTRUM-005 for their clinical characteristics and outcomes. In order to assess the robustness of the model, we conducted deterministic one-way sensitivity analyzes as well as probabilistic sensitivity analyzes. Subgroup analyzes were also conducted. Costs, quality-adjusted life-years (QALYs), life-years, incremental cost-effectiveness ratio (ICER), incremental net health benefits (INHB), and incremental net monetary benefits (INMB) were analyzed.

**Results:**

Based on the base-case analysis, serplulimab plus chemotherapy contributed to an increase in 0.826 life-years and 0.436 QALYs; an incremental cost of $52,331, yielded ICER of $120,149/QALY. Based on the willingness to pay (WTP) threshold of $37,669/QALY and $86,569/QALY, the INHB was −0.954 QALYs and − 0.169 QALYs and the INMB was -$35,924 and -$14,626, respectively. Based on the probabilistic sensitivity analysis results, serplulimab plus chemotherapy was unlikely to be cost-effective at a WTP threshold of $37,669/QALY and $86,569/QALY. One-way sensitivity analysis indicated that cost of serplulimab and body weight had the greatest impact on the model. Serplulimab plus chemotherapy could be cost-effective at a WTP threshold of $86,569/QALY when the cost of serplulimab was less than $5.24/mg or when the weight of the patient was less than 40.96 kg. Regardless of the WTP threshold at $37,669/QALY or $86,569. Serplulimab plus chemotherapy was not cost-effective in all subgroups.

**Conclusion:**

Serplulimab plus chemotherapy was not cost-effective, despite having a prior clinical benefical and a relative safety profile compared with chemotherapy. With the reduction in the price of serplulimab, ES-SCLC patients treated with serplulimab plus chemotherapy may be able to achieve a favorable cost-effectiveness rate.

## Introduction

There were approximately 1.8 million cancer-related deaths in 2020 related to lung cancer, making it the second most common cancer in the world, representing approximately 20 percent of all cancer deaths worldwide (1). There are approximately 15% of all lung cancers that are small cell lung cancers (SCLC) (2–4). When SCLC is initially diagnosed, patients with distant metastases or tumors make up 60–70% of advanced-stage patients that exceed the limits of a single radiation port (5, 6). The majority of patients were diagnosed with extensive-stage small-cell lung cancer (ES-SCLC), which is defined as cancer with distant metastases that cannot be treated with radiation (7). Extremely rapid progression has been associated with ES-SCLC, which is considered as a refractory cancer (8). In spite of the fact that most cases of ES-SCLC are initially susceptible to chemotherapy, local recurrences or distant metastases inevitably occur and no effective follow-up treatment is available, resulting in the prognosis is extremely poor for these patients (8, 9). It is therefore imperative that novel treatment regimens for ES-SCLC are developed as soon as possible.

Experimental treatment options with promising clinical outcomes are being investigated, including new immunotherapies and molecularly targeted agents. Patients with lung cancer benefit from immune checkpoint inhibitors (ICIs) that target the programmed death ligand-1 (PD-L1) or programmed death-1 (PD-1) axis, as well as the cytotoxic T-cell lymphocyte-4 (CTLA-4) (10). It has been fortunate that the addition of ICIs to chemotherapy has led to significant clinical beneficial in treating ES-SCLC in recent years, with improvements in treatment outcomes (11–13).

Serplulimab is a fully-humanized monoclonal antibody that targets the PD-1 receptor (14). In phase 3 randomized multicenter clinical trial, ASTRUM-005 (14) suggested that serplulimab plus chemotherapy was found to have improved clinical efficiency and safety as a first-line therapy for ES-SCLC when compared to chemotherapy alone. For serplulimab plus chemotherapy and chemotherapy, the median follow-up for overall survival (OS) was 15.4 and 10.9 months, respectively. There was a significant improvement in OS and progression-free survival (PFS) among patients with previously untreated ES-SCLC when treated with serplulimab and chemotherapy combination compared with chemotherapy alone. This suggests that serplulimab plus chemotherapy can be clinical efficiency as first-line treatment for ES-SCLC. In spite of this clinical beneficial, the high price of the drug has drawn attention. Currently, no cost-effectiveness analyzes have been published in which serplulimab plus chemotherapy is compared to chemotherapy for the therapy of ES-SCLC. Cost-effectiveness analysis is useful for ensuring that limited healthcare resources are distributed to physicians and decision-makers in the most optimal manner. Thus, the purpose of this study was to evaluate the cost-effectiveness of serplulimab combined with chemotherapy in treating ES-SCLC from the perspective of health care in China.

## Materials and methods

### Patients and intervention

Based on the Consolidated Health Economic Evaluation Reporting Standards (CHEERS), this study was conducted (15). In this study, the clinical, cost and utility data were obtaioned from the literature and open databases instead of data from individual patients, so no institutional review board approval or informed consent was required.

ASTRUM-005 randomized clinical trial provided hypotheses of target patients with ES-SCLC (14). Among the 585 patients who were enrolled in ASTRUM-005, the mean age of patients is 61.1 years (SD, 8.67 years), and 104 (17.8%) of included patients are women, and the detailed baseline characteristics of patients is shown in Supplementary Table S1. ES-SCLC was diagnosed histologically or cytologically in adults (aged 18 years or older) who had not previously received systemic treatment for ES-SCLC. Based on the Response Evaluation Criteria in Solid Tumors (RECIST), patients must have one or more measurable lesions, adequate organ function, an Eastern Cooperative Oncology Group Performance Status Scale score of 0 or 1, and a life expectancy of more than 12 weeks. Based on the ASTRUM-005 trial report (14), ES-SCLC patients assigned to the serplulimab plus chemotherapy arm received 4.5 mg/kg of serplulimab intravenously every 3 weeks in conjunction with chemotherapy. As part of the chemotherapy procedures, all patients received etoposide 100 mg/m^2^ on days 1, 2, and 3 as well as carboplatin within the area under the serum drug concentration-time curve (up to 750 mg) on day 1 of each cycle for a total of four cycles. When the disease progressed or unacceptable adverse events (AEs) occurred, subsequent therapies were received.

### Model structure

An economic evaluation was conducted in this study, and we performed a partitioned survival model using three mutually exclusive health states: PFS, progressive disease (PD), and death (16–19). Both treatment arms had a 10-year time horizon, and more than 98% of patients died during this period. The cycle length was 1 week. ASTRUM-005 trial results were used to determine the proportion of patients with OS and PFS in the model (14). Based on the area under the OS curve, the proportion of patients who were alive with OS, the proportion of patients who were alive with PFS, and the difference between the OS and PFS curves, the proportion of patients who had PD were evaluated.

### Clinical data inputs

Based on the results of the ASTRUM-005 trial (14), the OS and PFS curves were determined for patients with ES-SCLC receiving serplulimab plus chemotherapy and chemotherapy alone. Based on algorithm of Guyot et al., OS and PFS were extrapolated beyond the follow-up period of the trial (20). Our analysis was conducted using GetData Graph Digitizer version 2.26 (21) to extract data on time-to-survival, resulting in Kaplan–Meier survival curves for OS and PFS. Based on these data points, parametric survival functions were fit as follows: exponential, Weibull, gamma, log-normal, Gompertz, log-logistic, and generalized gamma distributions. Afterwards, the Akaike information criterion (AIC) and Bayesian information criterion (BIC) were used to select the best-fit parametric models. Parametric model of serplulimab plus chemotherapy and chemotherapy treatment is presented in Table 1, and goodness-of-fit results are presented in Supplementary Table S2. Log-logistic was selected to fit the OS and PFS K-M curves of serplulimab plus chemotherapy and PFS K-M curves of chemotherapy; lognormal was selected to fit the OS K-M curves of chemotherapy (Supplementary Figure S1). The key clinical input data are listed in Table 1.

**Table 1:** Key model inputs.

Parameter	Value (95% CI)	Distribution	Source
Survival model for serplulimab plus chemotherapy			
Log-logistic OS survival model of serplulimab plus chemotherapy^a^	γ = 1.7870 λ = 0.0124	ND	(14)
Log-logistic PFS survival model of serplulimab plus chemotherapy^a^	γ = 2.0490 λ = 0.0294	ND	(14)
Survival model for chemotherapy			
Lognormal OS survival model of chemotherapy^a^	μ = 4.1481 σ = 1.0719	ND	(14)
Log-logistic PFS survival model of chemotherapy^a^	γ = 2.5671 λ = 0.0448	ND	(14)
Cost input			
Drug costs per 1 mg			
Serplulimab^b^	8.32 (6.65 to 9.98)	Gamma	Local database
Etoposide	0.22 (0.17 to 0.26)	Gamma	Local database
Carboplatin	0.19 (0.15 to 0.23)	Gamma	Local database
Second-line treatment in serplulimab plus chemotherapy arm per cycle	33.28 (26.63 to 39.94)	Gamma	(14), Local database
Second-line treatment in chemotherapy arm per cycle	57.19 (45.75 to 68.63)	Gamma	(14), Local database
Cost of terminal care per patient^c^	2,596 (2077 to 3,116)	Gamma	(22)
Disease costs per cycle^d^			
PFS	185 (148 to 222)	Gamma	(22)
PD	552 (441 to 662)	Gamma	(22)
Cost of managing AEs (grade ≥ 3)			
Serplulimab plus chemotherapy	1,562 (1,250 to 1874)	Gamma	(23–25)
Chemotherapy	1,697 (1,358 to 2036)	Gamma	(23–25)
Cost of drug administration per unit	19.11 (15.288 to 22.932)	Gamma	(26)
Health utilities			
Disease status utility per year			
PFS	0.673 (0.538 to 0.808)	Beta	(28, 29)
PD	0.473 (0.378 to 0.568)	Beta	(28, 29)
Death	0	NA	
Disutility due to AEs (grade ≥ 3)			
Serplulimab plus chemotherapy	0.050 (0.040 to 0.060)	Beta	(29–31)
Chemotherapy	0.053 (0.042 to 0.064)	Beta	(29–31)
Other model inputs			
Body surface area, m^2^	1.80 (1.44 to 2.16)	Normal	(32, 33)
Body weight, kg	65 (50 to 90)	Normal	(32, 33)
Creatinine clearance rate, ml/min/1.73m^2^	90 (80 to 100)	Normal	(32)

PFS, progression-free survival; OS, overall survival; HR, hazard ratio; ND, not determined; PD, progressed disease; AEs, adverse events. ^a^Only expected values are presented for these survival model parameters. ^b^Treatment with serplulimab continued until disease progression or unacceptable toxicity. ^c^Overall total cost per patient regardless of treatment duration. ^d^These costs were assumed to be continued until the health state transitioned.

### Cost

Costs associated with direct medical treatment were calculated, including the cost of drugs, costs associated with the health condition of patients, costs associated with disease supportive care, costs associated with terminal care, and costs associated with AEs (Table 1). Databases of local hospitals were used to determine the cost of acquiring drugs. It was estimated that the disease supportive care costs for patients with PFS and PD would be $185 and $552 per cycle, respectively (22). Approximately 44% of patients receiving serplulimab plus chemotherapy and 43% of patients receiving chemotherapy received second-line treatments in the ASTRUM-005 trial (14). The costs related to management of grade ≥ 3 AEs were obtained from the literature (Supplementary Table S3) (23–25). Cost of drug administration was obtained from the literature (26). The costs have been inflated to 2021 monetary values using the Medical-Care Inflation data from Tom’s Inflation Calculator (27), which are displayed in Table 1.

### Effectiveness

The health utility scores were evaluated to a scale ranging from zero (death) to one (perfect health). Due to the fact that health utilities for PFS and PD were not evaluated in ASTRUM-005, we selected published health utility (28, 29). Based on previously published literature evaluated patients with ES-SCLC, we selected the health utilities of PFS and PD were 0.673 and 0.473, respectively (28, 29). From the literature, we also obtained the disutility values related to AEs (29–31).

### Base case analysis

In this study, we calculated the incremental cost-effectiveness ratio (ICER) by the incremental cost dividing additional quality-adjusted life year (QALY). For purposes of calculating the dose of chemotherapy and ICIs, we assumed that the average body surface area (BSA), weight, and creatinine clearance rate (CCR) were 1.80 m^2^, 65 kg, and 90 mL/min/1.73 m^2^, respectively (32, 33). We calculated the ICERs with two willingness to pay (WTP) thresholds based on the imbalanced economic development of different socioeconomic regions in China: three times the *per capita* GDP of China in 2021 ($37,669/QALY) for general regions and three times the *per capita* GDP of Beijing city in 2021 ($86,569/QALY) for affluent regions (34). Costs and utility outcomes were discounted annually at a rate of 5%. Furthermore, we evaluated the incremental net health benefits (INHB) as well as the incremental net monetary benefits (INMB) (17–19). The INHB and INMB are calculated based on the following formulas: $INHB\left(\lambda \right)=\left(\mu_{Esc}-\mu_{Ec}\right)-\frac{\mu_{\csc}-\mu_{Cc}}{\lambda }=\Delta E-\Delta C/\lambda$ and, $INMB\left(\lambda \right)=\left(\mu_{Esc}-\mu_{Ec}\right)\times \lambda -\left(\mu_{\csc}-\mu_{Cc}\right)=\Delta E\times \lambda -\Delta C$, where *μ*_Esc_ and *μ*_Ec_ are the effectiveness of serplulimab plus chemotherapy and chemotherapy, respectively; *μ*_Csc_, *μ*_Cc_ and are the costs of serplulimab plus chemotherapy and chemotherapy, respectively; and λ is the WTP threshold.

### Sensitivity analyzes

One-way sensitivity analysis was performed in this study to identify variables that are sensitive to the results. Various variables, such as costs and utilities, were subjected to one-way sensitivity analyzes, and the uncertainty for each variable was evaluated based on 95% confidence intervals (CIs) published in the literature or estimated by assuming a 20% variation from the fundamental variable (Table 1). In addition, we conducted a probabilistic sensitivity analysis using Monte Carlo simulations with 10,000 iterations. The parameters determined a suitable distribution. For the cost, hazard ratios (HRs), proportion, probability, and preference value variables, beta, log-normal, and gamma distributions were assigned. In order to reflect the possibility of serplulimab plus chemotherapy or chemotherapy being beneficial at various levels of WTP/QALY gains, a cost-effectiveness acceptability curve was constructed.

### Subgroup analyzes

To explore the impact of different patient characteristics on the outcomes, subgroup analyzes were performed. Different HR for OS for the different subgroups obtained from ASTRUM-005, subgroup analyzes were developed for each subgroup, including patients’ age, gender, race, baseline Eastern Cooperative Oncology Group (ECOG) performance status scale score, smoking history, brain metastases, and PD-L1 expression level (14). We conducted our statistical analyzes in R, version 4.0.5, 2021 (R Foundation for Statistical Computing) using the hesim and heemod packages.

## Results

### Base case analysis

Based on the standard case analysis of patients with ES-SCLC, serplulimab plus chemotherapy resulted in an increase in QALYs of 0.436, an increase in overall life years of 0.826, and an increase in costs of $52,331 over the chemotherapy arm alone. It is estimated that the ICER was $120,149/QALY. Furthermore, the INHB of serplulimab plus chemotherapy were − 0.954 QALYs and − 0.169 QALYs; and INMB were -$35,924 and -$14,626, respectively, at a $37,669/QALY and $86,569 WTP threshold compared with chemotherapy (Table 2).

**Table 2:** Summary of cost and outcome results in the base-case analysis.

Factor	Serplulimab plus Chemotherapy	Chemotherapy	Incremental change
Cost, $			
Drug^a^	44,449	4,719	39,730
Nondrug^b^	46,210	33,608	12,602
Overall	90,659	38,327	52,331
Life-years			
Progression-free	0.967	0.498	0.469
Overall	2.313	1.487	0.826
QALYs	1.207	0.771	0.436
ICERs, $			
Per life-year	NA	NA	63,345
Per QALY	NA	NA	120,149
INHB, QALY, at WTP threshold 37,669^a^	NA	NA	−0.954
INMB, $, at WTP threshold 37,669^a^	NA	NA	−35,924
INHB, QALY, at WTP threshold 86,569^a^	NA	NA	−0.169
INMB, $, at WTP threshold 86,569^a^	NA	NA	−14,626

ICER, incremental cost-effectiveness ratio; INHB, incremental net health benefit; INMB, incremental net monetary benefit; NA, not applicable; QALYs, quality-adjusted life years. ^a^Compared with chemotherapy.

^b^Nondrug cost includes the costs of adverse event management, subsequent best supportive care per patient, and follow-up care covering physician monitors, drug administration, and terminal care.

### Sensitivity analysis

**Figure 1:**
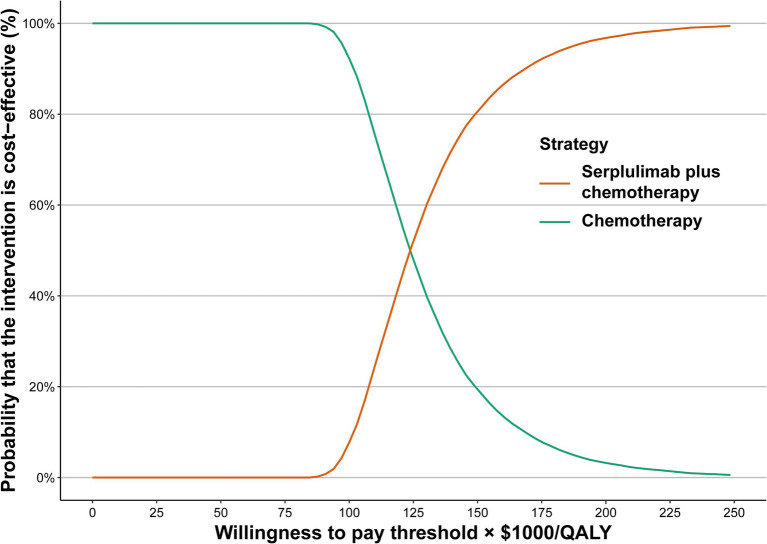
Acceptability curves for the choice of serplulimab plus chemotherapy treatment strategies at different WTP thresholds in patients with extensive-stage small cell lung cancer. WTP, willingness to pay.

One-way sensitivity analysis suggested that the body weight of patients, the HR of serplulimab plus chemotherapy versus chemotherapy, the cost of serplulimab, as well as their utility for both PFS and PD, were the primary drivers of the model outcome (Supplementary Figure S2). Additionally, we evaluated the relevance of these key variables with regard to the ICER when comparing serplulimab plus chemotherapy with chemotherapy. Serplulimab plus chemotherapy could be considered as a cost-effective option when the price of serplulimab was less than $0.76 per mg and $5.24 per mg at WTP thresholds of $37,669/QALY and $86,569/QALY, respectively. (Supplementary Figure S3). Serplulimab plus chemotherapy, however, is cost-effective when patients weigh less than 40.96 kg at a threshold WTP of $86,569/QALY. Cost-effectiveness acceptability curves were used to display the results of the probabilistic sensitivity analysis (Figure 1). As WTP thresholds increased, it became more likely that serplulimab plus chemotherapy would be cost-effective. Comparison of serplulimab plus chemotherapy with chemotherapy indicated that the probability of serplulimab plus chemotherapy being considered cost-effective was only 0 and 0.04%, respectively, at WTP thresholds of $37,669/QALY and $86,569/QALY.

### Subgroup analysis

Variations in OS HRs were used to perform the subgroup analysis. It was found that serplulimab plus chemotherapy resulted in significantly different OS than chemotherapy alone in the majority of the subgroups (Table 3). Subgroup analysis suggested that serplulimab plus chemotherapy had a relatively higher probability of being considered cost-effective according to the ECOG performance status scale score, not evaluable or not available of PD-L1 expression level subgroup at an $86,569/QALY WTP threshold (Table 3). However, serplulimab plus chemotherapy may not have a probability of cost-effective in all of the subgroups, regardless of whether the WTP threshold was selected at $37,669/QALY or $86,569.

**Table 3:** Summary of subgroup analyzes obtained by varying the hazard ratios (HRs) for overall survival.

Subgroup	Unstratified hazard ratio for OS (95% CI)	Change in cost, $^a^	Change in QALYs^a^	ICER, $/QALY	Cost-effectiveness probability of serplulimab plus chemotherapy, %		INHB, QALY	
					WTP of $37,669/QALY	WTP of $86,569/QALY	WTP of $37,669/QALY	WTP of $86,569/QALY
Age, years								
<65	0.62 (0.45–0.86)	52,898	0.445	118,885	0	0.18	−0.959	−0.166
≥65	0.60 (0.40–0.89)	54,028	0.464	116,518	0	0.49	−0.971	−0.160
Sex								
Male	0.64 (0.48–0.84)	51,764	0.426	121,470	0	0.04	−0.948	−0.172
Female	0.57 (0.30–1.06)	55,715	0.492	113,321	0	1.15	−0.987	−0.152
Race								
Asian	0.58 (0.43–0.79)	55,154	0.482	114,343	0	0.78	−0.982	−0.155
Non-Asian	0.70 (0.43–1.13)	48,343	0.369	130,859	0	0	−0.914	−0.189
Baseline ECOG performance status scale score								
0	0.44 (0.23–0.84)	62,885	0.611	102,999	0	9.29	−1.059	−0.116
1	0.65 (0.49–0.86)	51,195	0.417	122,853	0	0.01	−0.942	−0.175
Smoking history								
Never	0.75 (0.42–1.33)	45,480	0.322	141,261	0	0	−0.885	−0.203
Current	0.61 (0.36–1.02)	53,463	0.454	117,676	0	0.25	−0.965	−0.163
Former	0.59 (0.42–0.83)	54,592	0.473	115,408	0	0.61	−0.976	−0.158
Brain metastases								
No	0.62 (0.47–0.82)	52,898	0.445	118,885	0	0.11	−0.959	−0.166
Yes	0.61 (0.33–1.13)	53,463	0.454	117,676	0	0.29	−0.965	−0.163
PD-L1 expression level								
Tumor proportion score < 1%	0.58 (0.44–0.76)	55,154	0.482	114,343	0	0.71	−0.982	−0.155
Tumor proportion score ≥ 1%	0.92 (0.44–1.89)	35,776	0.161	222,137	0	0	−0.789	−0.252
Not evaluable or not available	0.42 (0.10–1.72)	63,967	0.628	101,780	0	10.35	−1.070	−0.110

ECOG, Eastern Cooperative Oncology Group; HR, hazard ratio; ICER, incremental cost-effectiveness ratio; OS, overall survival; PD-L1, programmed cell death ligand 1; QALY, quality-adjusted life-year; WTP, willingness to pay. ^a^HR for OS represents the HR of serplulimab plus chemotherapy versus chemotherapy for OS; change in cost and change in QALYs represent the results of serplulimab plus chemotherapy minus chemotherapy.

## Discussion

The purpose of this study was to assess the cost-effectiveness of serplulimab plus chemotherapy versus chemotherapy for the therapy of ES-SCLC, and the results of this study suggested that compared with chemotherapy, serplulimab plus chemotherapy was related to incremental survival of 0.436 QALYs and incremental cost of $52,331. The calculated ICER was $120,149/QALY. The results of the one-way sensitivity analysis showed that body weight, HR for OS of serplulimab plus chemotherapy versus chemotherapy, and cost of serplulimab were the most sensitive factors on ICER, revealed that the choice between serplulimab plus chemotherapy and chemotherapy can be altered based on body weight and cost of serplulimab. With serplulimab priced reducing to $0.76/mg and $5.24/mg, serplulimab plus chemotherapy was cost-effective at a WTP threshold of $37,669/QALY and $86,569/QALY respectively, or when the weight of patients was less than 40.96 kg at a WTP threshold of $86,569/QALY. Serplulimab plus chemotherapy for the treatment of ES-SCLC was unlikely to be a cost-effective treatment option based on the results of this study at a WTP threshold of $37,669/QALY and $86,569/QALY. The results of this study are robust according to one-way sensitivity analysis and probabilistic sensitivity analysis. Cost-effectiveness acceptability curve revealed that serplulimab plus chemotherapy was not a cost-effective option when the WTP threshold was $37,669/QALY or $86,569/QALY, respectively.

Until now, this is the first study to perform a cost-effectiveness analysis comparing serplulimab and chemotherapy as first-line therapy for ES-SCLC. In recent years, ICIs have been approved for the treatment of ES-SCLC. Prices for ICIs are often high because of the high costs associated with their development. Therefore, ICI is not as cost-effective option as indicated in the published study. In a previously published study, the cost-effectiveness of atezolizumab plus chemotherapy compared to chemotherapy for the treatment of ES-SCLC from the perspective of the Chinese healthcare system was examined. In comparison with chemotherapy alone, the ICER for atezolizumab plus chemotherapy was $489,013/QALY (35). According to another study, comparing durvalumab plus chemotherapy with chemotherapy alone for the therapy of ES-SCLC in the Chinese healthcare system, durvalumab plus chemotherapy led to an ICER of $302,051/QALY (36). Another two studies analyzed pembrolizumab plus chemotherapy compared to chemotherapy for the therapy of ES-SCLC the US payer perspective suggested that pembrolizumab plus chemotherapy related to an ICER of $334,373/QALY (37) and $647,509/QALY (38) compared to chemotherapy alone. Studies above showed that ICIs were not a cost-effective therapy option when compared to chemotherapy. Despite this, adebrelimab was evaluated as a first-line treatment for advanced ES-SCLC, and it was found to be a cost-effective option than chemotherapy, with an ICER of $26,914/QALY from the perspective of the Chinese healthcare system (39). Previously published study evaluated the cost-effectiveness of serplulimab plus chemotherapy for ES-SCLC (40). It revealed that serplulimab plus chemotherapy was related to an increase of 0.332 QALYs and an additional cost of $4,008, and yielded corresponding ICER was $12,077/QALY. Compared with previous study, several differences were noted in this study and this may be related to the difference of ICER. First, in this study, we adopted partitioned survival model to evaluate the cost-effectiveness of serplulimab plus chemotherapy for ES-SCLC, and Markov models was used in previous published study. In a partitioned survival model, health state occupancy is estimated directly from the area under the relevant survival curve. Partitioned survival models differ from state transition models (STM) such as Markov models, as they do not include a structural link between intermediate clinical endpoints (e.g., disease progression) and survival. Partitioned survival models directly consider clinical trial endpoints and can be developed without access to individual patient data. On the other hand, partitioned survival models and STMs can produce substantively different survival extrapolations and extrapolations from STMs are heavily influenced by specification of the underlying survival models (41). In a partitioned survival model, OS extrapolation reflects only the OS evidence and not PFS, whereas in a STM, OS extrapolation is influenced by the model structure and each transition probability estimate (41). Second, our study also included the cost of second-line treatment. Due to the high price of serplulimab in the base-case analysis, the fluctuation in subsequent treatment proportion of using serplulimab may influence the ICER. Third, in this study, we obtained the price of drugs from the local database of several hospital, and the public hospitals in China implemented a policy that the selling price of drugs was in accordance with the purchasing price of drugs. The cost of drugs can be reflected as the real situation in China. Fourth, in this study, we also performed subgroup analysis. It may be helpful for both patients and physicians to clinical evidence on the economic status of subgroups when making clinical decisions. Lastly, in this study, we also calculated INHB and INMB, in a resource-constrained health care system, health care costs really represent the health outcomes for other patients with competing claims on health care resources; therefore, decisions based on economic evaluation are really about identifying the alternative which offers the greatest INHB or INMB overall (42, 43). Furthermore, the National Healthcare Security Administration (NHSA) in China has made great efforts to negotiate drug prices with pharmaceutical companies, resulting in a reduction of 30–70% in the price of many anticancer drugs, including ICIs (44). Serplulimab is unlikely to experience a trend of rising prices for this reason.

There are several advantages to this study. First, this study combined the recently published randomized clinical trial with a partitioned survival model to evaluate the cost-effectiveness of serplulimab plus chemotherapy for the therapy of ES-SCLC. Second, the sensitive factors have been evaluated, and the results of our study are robust. There is no need to adopt a fixed dose instead of a weight-based regimen, and the dosing regimen should be tailored to the individual patient. In order to make a appropriate clinical decision, it is recommended that both the WTP threshold and the weight of the patient be considered. Third, it may be beneficial for both patients and physicians to have information on the economic status of subgroups when making clinical decisions.

This study had some limitations. First, the model outputs may have been uncertain due to the use of parametric distributions to fit the published K-M OS and PFS data, which leaded to health outcomes beyond the follow-up time of ASTRUM-005 trial. According to the results of the sensitivity analysis, it appears that this limitation may not be a major factor in determining this finding. Secondly, it is important to note that we only considered the virtual cost of serplulimab in the local database. This is without taking into account the different health insurance plans and discounts offered. It is therefore recommended that real clinical data be used to evaluated values following the approval of serplulimab for sales in mainland China. There are, however, differences in the health insurance policies and company discounts in different regions of China, so it is inaccurate to recalculate the values for each region. We also evaluated the sensitive factors, including the cost of serplulimab, and detailed results have been shown. The aim of this study is to provide drug pricing and medical insurance companies with a decision-making reference. Last, the majority of included patients in the study were Chinese, one-third of patients included in the study were non-Asian, and the utility values were obtained from a foreign study, which might result in bias in the analysis. The ICER far exceeded the WTP threshold in China and the probabilistic sensitivity analysis demonstrated robust results, even though the medium impact was showed in one-way sensitivity analysis. In spite of these limitations, we believe that our analysis provides a good representation of the clinical conditions of ES-SCLC in China, and we hope that our findings will be useful to Chinese decision-makers.

## Conclusion

From the perspective of the Chinese health-care system, serplulimab plus chemotherapy as the first-line therapy for ES-SCLC is unlikely to be a cost-effective strategy. The cost-effectiveness of this treatment could be changed if the price of serplulimab were reduced and consideration was given to the weight of the patient.

## Data availability statement

The original contributions presented in the study are included in the article/Supplementary material, further inquiries can be directed to the corresponding author.

## Author contributions

XL conceived and designed the study, constructed the model, did the literature search and the acquisition of data, analyzed the data and revised the manuscript. XC conceived and designed the study, acquired the funding, did the acquisition of data, revised the manuscript. HL revised the manuscript, technical and material support, and approved the final version of the manuscript. YL participated in study concept and design, constructed the model, did the literature search and the acquisition of data, analyzed the data and drafted the manuscript. All authors contributed to the article and approved the submitted version.

## Funding

This work was supported by the National Natural Science Foundation of China (No. 82160763).

## Conflict of interest

The authors declare that the research was conducted in the absence of any commercial or financial relationships that could be construed as a potential conflict of interest.

## Publisher’s note

All claims expressed in this article are solely those of the authors and do not necessarily represent those of their affiliated organizations, or those of the publisher, the editors and the reviewers. Any product that may be evaluated in this article, or claim that may be made by its manufacturer, is not guaranteed or endorsed by the publisher.
