# Supplementary material for: Cost-effectiveness analysis of first-line serplulimab combined with chemotherapy for extensive-stage small cell lung cancer
Source: Front Public Health. 2023 Aug 31;11:1156427. doi: 10.3389/fpubh.2023.1156427 (PMC10505963; doi:10.3389/fpubh.2023.1156427)
Supplement: Supplementary file 1 [file Data_Sheet_1.docx]

**Supplementary Content**

**Supplementary Figure 1.** Model Fitting Analysis

**Supplementary Figure 2.** Tornado Diagram of One-Way Sensitivity Analyses

**Supplementary Figure 3.** Impacts of Key Factors on Incremental Cost-effectiveness Ratio

**Supplementary Table 1.** Baseline Characteristics of Patients in ASTRUM-005

**Supplementary Table 2.** Akaike Information Criterion and Bayesian Information Criterion Values from Each Survival Model

**Supplementary Table 3.** Associated Costs and Disutility of Grade ≥ 3 Treatment-Related Adverse Events

**Supplementary Figure 1.** Model Fitting Analysis

To obtain the best model fit, the following investigations were carried out using serplulimab plus chemotherapy or chemotherapy as the model fit baseline, respectively. Based on values of AIC and BIC (Supplementary Table 2), log-logistic was used to fit the OS and PFS K-M curves of serplulimab plus chemotherapy and PFS K-M curves of chemotherapy; lognormal was used to fit the OS K-M curves of chemotherapy.

(A) Model-fitted versus original K-M curves for serplulimab plus chemotherapy.


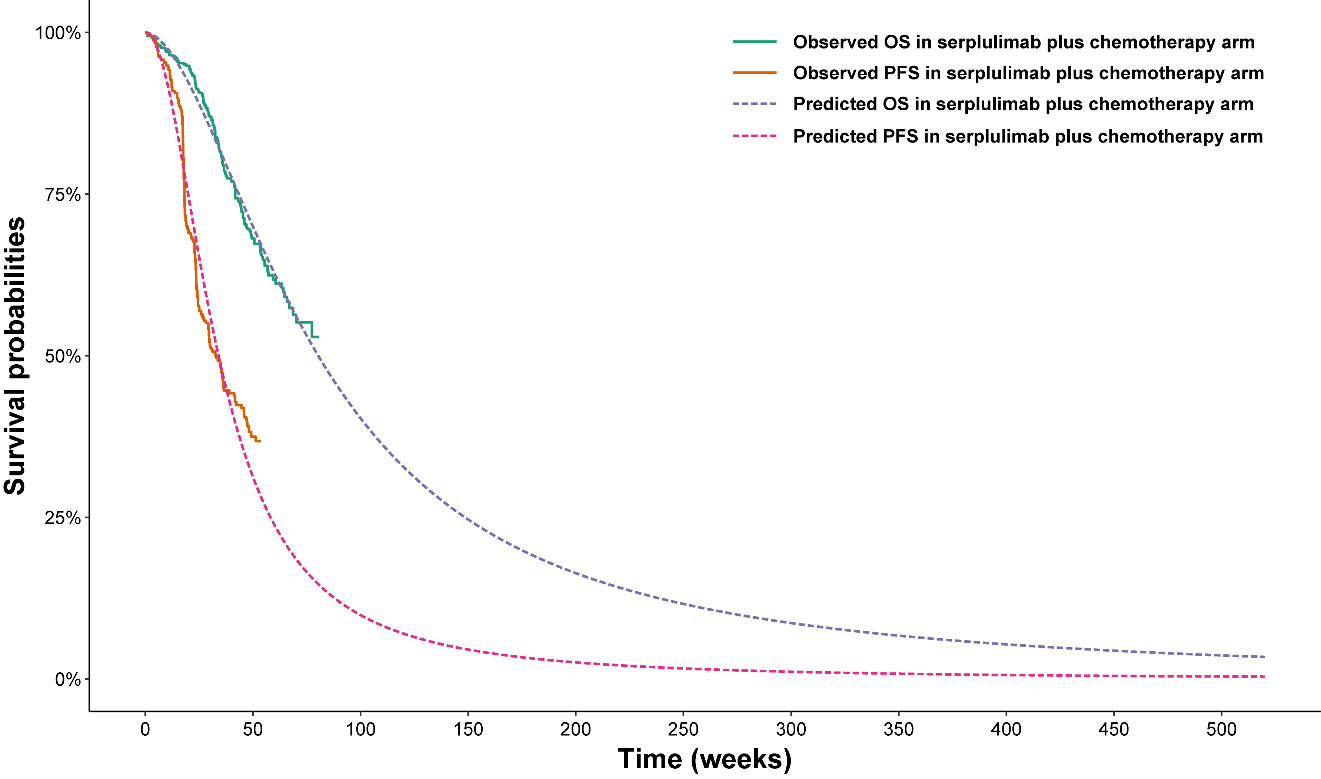


(B) Model-fitted versus original K-M curves for chemotherapy.


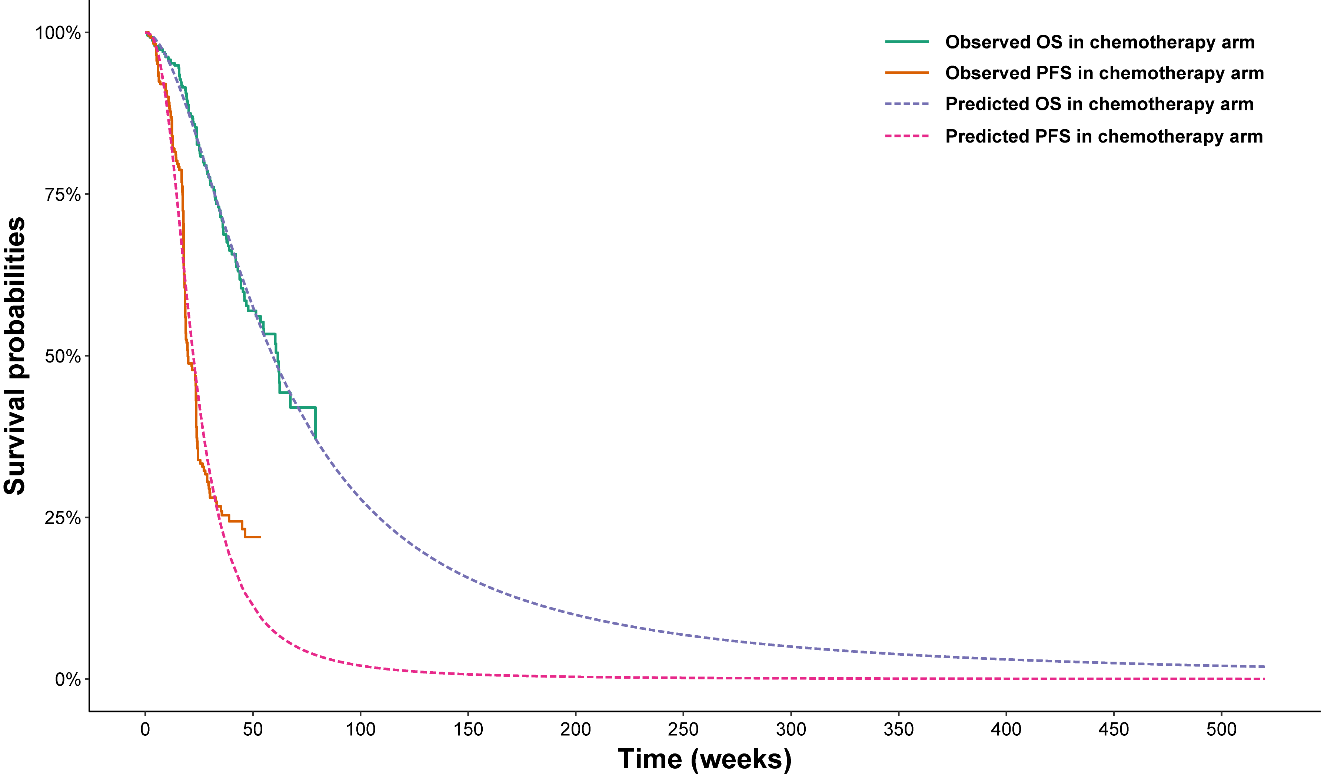


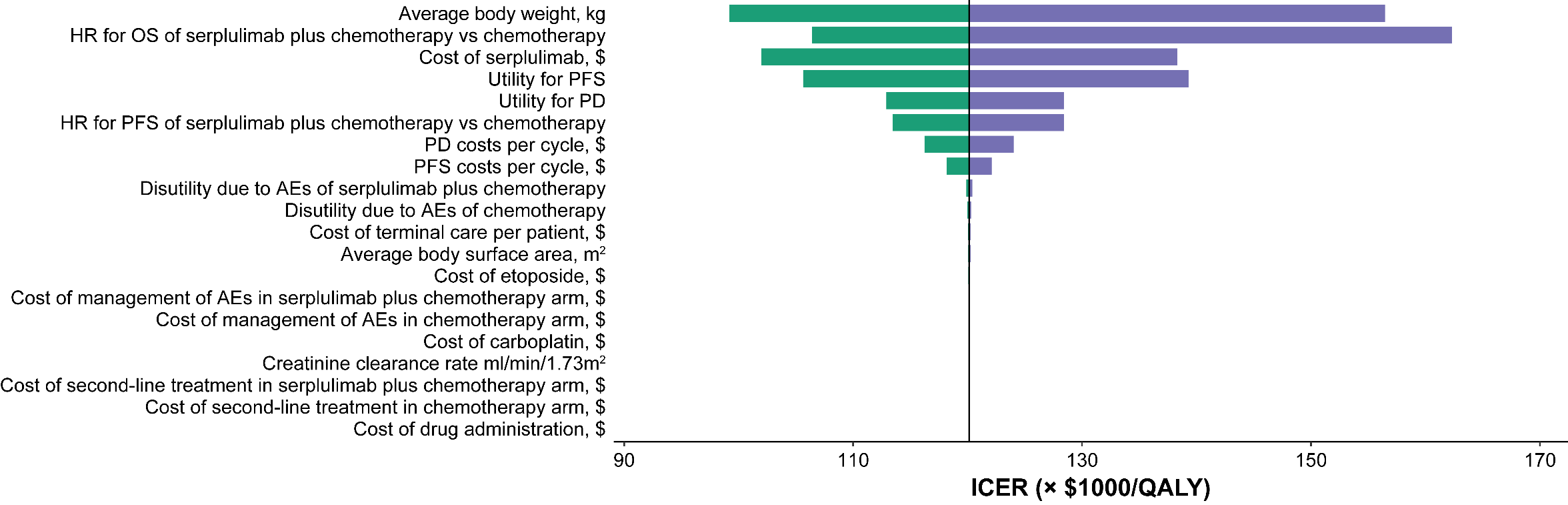


**Supplementary Figure 2.** Tornado Diagram of One-Way Sensitivity Analyses. OS, overall survival; HR, hazard ratio; PD, progressed disease; PFS, progression-free survival; AEs, adverse events.

**Supplementary Figure 3.** Impacts of Key Factors on Incremental Cost-effectiveness Ratio

The diagrams show the impacts of key factors on the incremental cost-effectiveness ratio (serplulimab plus chemotherapy versus chemotherapy) for the treatment of extensive-stage small cell lung cancer. (A) represents the impacts of the cost of serplulimab; (B) represents the weight of patients. ICER: Incremental cost-effectiveness ratio; QALY: Quality-adjusted life year.

(A) represents the impacts of the cost of serplulimab


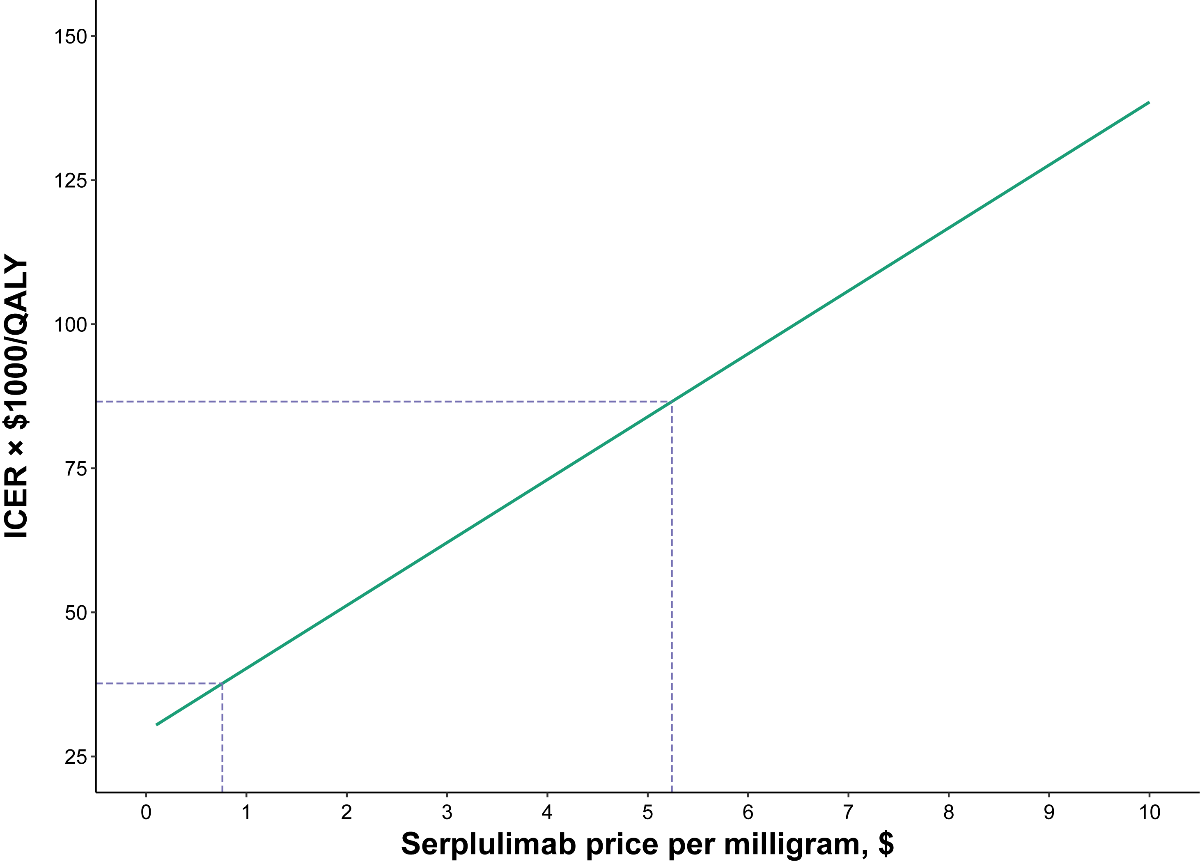


(B) represents the impacts of the weight of patients


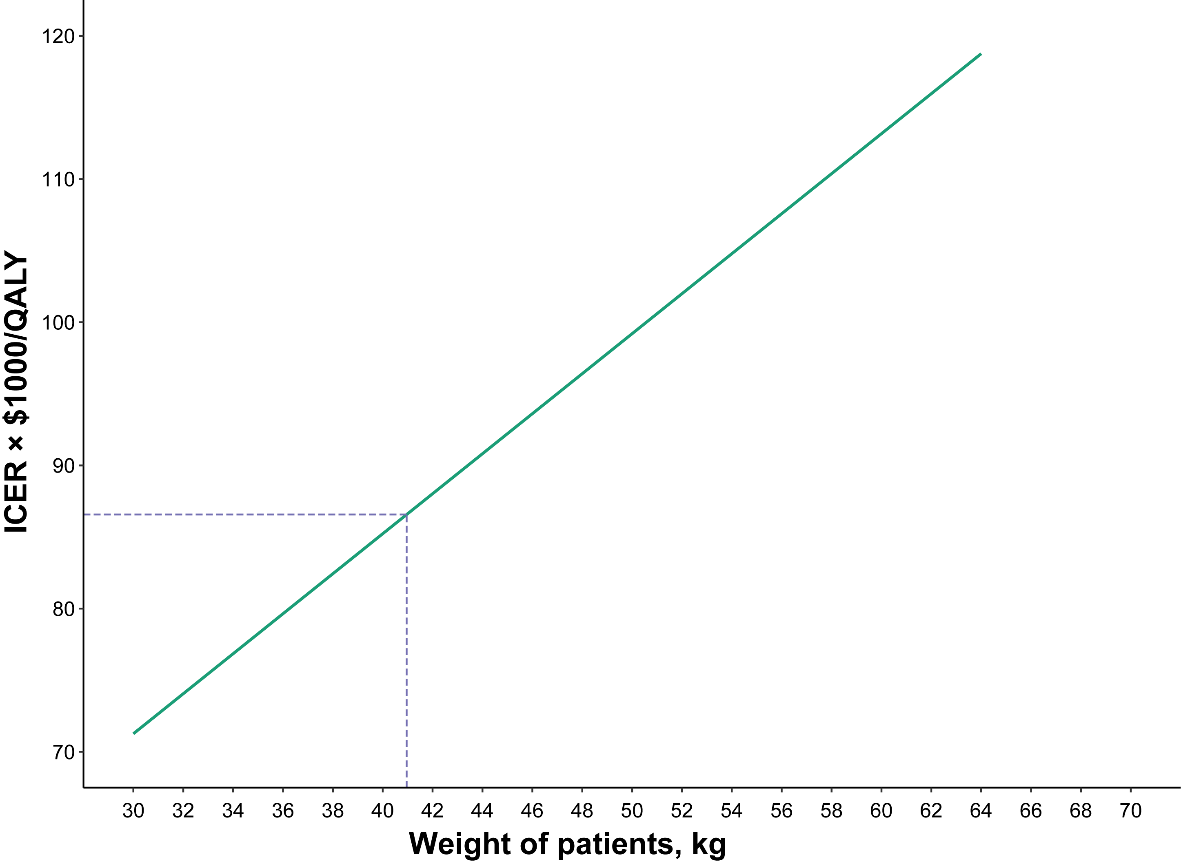


**Supplementary Table 1.** Baseline Characteristics of Patients in ASTRUM-005^a^

|  | **Serplulimab plus chemotherapy (n = 389), No. (%)** | **Chemotherapy (n = 196), No. (%)** |
| --- | --- | --- |
| Age, median (range), years | 63 (28-76) | 62 (31-83) |
| Aged <65 years | 235 (60.4) | 119 (60.7) |
| Sex |  |  |
| Male | 317 (81.5) | 164 (83.7) |
| Female | 72 (18.5) | 32 (16.3) |
| Race |  |  |
| Asian | 262 (67.4) | 139 (70.9) |
| Non-Asian | 127 (32.6) | 57 (29.1) |
| Eastern Cooperative Oncology Group Performance Status Scale score | | |
| 0 | 71 (18.3) | 32 (16.3) |
| 1 | 318 (81.7) | 164 (83.7) |
| Smoking history |  |  |
| Never | 81 (20.8) | 35 (17.9) |
| Current | 102 (26.2) | 48 (24.5) |
| Former | 206 (53.0) | 113 (57.7) |
| Size of target lesions, median (range), mm in diameter | 117.7 (13.8-323.7) | 120.5 (14.5-269.6) |
| Type of metastases |  |  |
| Brain | 50 (12.9) | 28 (14.3) |
| Liver | 99 (25.4) | 51 (26.0) |
| Programmed cell death ligand 1 expression level, No./total (%) | | |
| Tumor proportion score <1% | 317/379 (83.6) | 152/186 (81.7) |
| Tumor proportion score ≥1% | 62/379 (16.4) | 34/186 (18.3) |
| Previous cancer treatment |  |  |
| Chemotherapy | 9 (2.3) | 3 (1.5) |
| Other | 1 (0.3) | 2 (1.0) |

^a^Reference: Cheng Y, Han L, Wu L, Chen J, Sun H, Wen G, et al. Effect of first-line Serplulimab vs placebo added to chemotherapy on survival in patients with extensive-stage small cell lung Cancer: the ASTRUM-005 randomized clinical trial. JAMA. (2022) 328:1223–32. doi: 10.1001/jama.2022.16464

**Supplementary Table 2.** Akaike Information Criterion and Bayesian Information Criterion Values from Each Survival Model. AIC, Akaike information criterion; BIC, Bayesian Information Criterion; OS, overall survival; PFS, progression-free survival.

| **Strategies** | **Distributions** | **Parameters** | **est** | **se** | **L95%** | **U95%** | **AIC** | **BIC** |
| --- | --- | --- | --- | --- | --- | --- | --- | --- |
| **Results of OS** | | | | | | | | |
| **Serplulimab plus chemotherapy** | Exponential | rate | 0.0070 | 0.0006 | 0.0058 | 0.0084 | 1397.687 | 1401.651 |
|  | Weibull | shape | 1.5931 | 0.1291 | 1.3592 | 1.8673 | 1373.028 | 1380.955 |
|  |  | scale | 0.0007 | 0.0004 | 0.0002 | 0.0019 |  |  |
|  | Gamma | shape | 1.7959 | 0.1944 | 1.4525 | 2.2204 | 1373.733 | 1381.66 |
|  |  | rate | 0.0186 | 0.0033 | 0.0132 | 0.0263 |  |  |
|  | Lognormal | meanlog | 4.5340 | 0.0996 | 4.3388 | 4.7292 | 1388.43 | 1396.357 |
|  |  | sdlog | 1.1608 | 0.0828 | 1.0094 | 1.3350 |  |  |
|  | Gompertz | shape | 0.0234 | 0.0048 | 0.0140 | 0.0328 | 1376.9 | 1384.828 |
|  |  | rate | 0.0035 | 0.0007 | 0.0024 | 0.0050 |  |  |
|  | Log-logistic | shape | 1.7870 | 0.1444 | 1.5253 | 2.0936 | **1372.5** | **1380.427** |
|  |  | scale | 80.3539 | 6.2029 | 69.0714 | 93.4793 |  |  |
|  | Generalized gamma | mu | 4.5742 | 0.0781 | 4.4211 | 4.7274 | 1374.437 | 1386.328 |
|  |  | sigma | 0.5956 | 0.1394 | 0.3765 | 0.9421 |  |  |
|  |  | Q | 1.0850 | 0.3565 | 0.3863 | 1.7836 |  |  |
| **Chemotherapy** | Exponential | rate | 0.0103 | 0.0012 | 0.0082 | 0.0128 | 871.997 | 875.275 |
|  | Weibull | shape | 1.5375 | 0.1486 | 1.2721 | 1.8582 | 857.624 | 864.18 |
|  |  | scale | 0.0013 | 0.0008 | 0.0004 | 0.0042 |  |  |
|  | Gamma | shape | 1.8009 | 0.2447 | 1.3800 | 2.3503 | 857.446 | 864.002 |
|  |  | rate | 0.0250 | 0.0051 | 0.0167 | 0.0374 |  |  |
|  | Lognormal | meanlog | 4.1481 | 0.1076 | 3.9371 | 4.3591 | **856.905** | **863.461** |
|  |  | sdlog | 1.0719 | 0.0925 | 0.9051 | 1.2694 |  |  |
|  | Gompertz | shape | 0.0201 | 0.0059 | 0.0086 | 0.0316 | 863.02 | 869.577 |
|  |  | rate | 0.0060 | 0.0013 | 0.0039 | 0.0091 |  |  |
|  | Log-logistic | shape | 1.8102 | 0.1757 | 1.4967 | 2.1894 | 863.867 | 870.424 |
|  |  | scale | 59.1827 | 5.2559 | 49.7280 | 70.4350 |  |  |
|  | Generalized gamma | mu | 4.2869 | 0.1034 | 4.0842 | 4.4896 | 859.394 | 869.228 |
|  |  | sigma | 0.7137 | 0.1461 | 0.4778 | 1.0659 |  |  |
|  |  | Q | 0.8222 | 0.3485 | 0.1392 | 1.5052 |  |  |
| **Results of PFS** | | | | | | | | |
| **Serplulimab plus chemotherapy** | Exponential | rate | 0.0176 | 0.0014 | 0.0151 | 0.0205 | 1685.232 | 1689.196 |
|  | Weibull | shape | 1.5795 | 0.0983 | 1.3982 | 1.7844 | 1643.653 | 1651.58 |
|  |  | scale | 0.0024 | 0.0008 | 0.0012 | 0.0048 |  |  |
|  | Gamma | shape | 2.0918 | 0.1976 | 1.7383 | 2.5173 | 1634.798 | 1642.725 |
|  |  | rate | 0.0497 | 0.0065 | 0.0384 | 0.0644 |  |  |
|  | Lognormal | meanlog | 3.5676 | 0.0600 | 3.4501 | 3.6851 | 1628.205 | 1636.132 |
|  |  | sdlog | 0.8852 | 0.0511 | 0.7905 | 0.9913 |  |  |
|  | Gompertz | shape | 0.0212 | 0.0054 | 0.0107 | 0.0317 | 1672.352 | 1680.279 |
|  |  | rate | 0.0119 | 0.0016 | 0.0092 | 0.0155 |  |  |
|  | Log-logistic | shape | 2.0490 | 0.1311 | 1.8075 | 2.3229 | **1624.285** | **1632.212** |
|  |  | scale | 34.0545 | 1.8440 | 30.6255 | 37.8675 |  |  |
|  | Generalized gamma | mu | 3.6083 | 0.0791 | 3.4533 | 3.7633 | 1629.632 | 1641.523 |
|  |  | sigma | 0.8449 | 0.0721 | 0.7147 | 0.9988 |  |  |
|  |  | Q | 0.1533 | 0.2017 | -0.2420 | 0.5486 |  |  |
| **Chemotherapy** | Exponential | rate | 0.0294 | 0.0028 | 0.0244 | 0.0355 | 988.484 | 991.762 |
|  | Weibull | shape | 1.7944 | 0.1331 | 1.5515 | 2.0752 | 944.617 | 951.173 |
|  |  | scale | 0.0023 | 0.0010 | 0.0010 | 0.0056 |  |  |
|  | Gamma | shape | 2.6903 | 0.3296 | 2.1160 | 3.4205 | 937.947 | 944.503 |
|  |  | rate | 0.1020 | 0.0156 | 0.0755 | 0.1376 |  |  |
|  | Lognormal | meanlog | 3.1217 | 0.0622 | 2.9997 | 3.2437 | 939.049 | 945.605 |
|  |  | sdlog | 0.7358 | 0.0518 | 0.6409 | 0.8447 |  |  |
|  | Gompertz | shape | 0.0371 | 0.0076 | 0.0222 | 0.0521 | 970.223 | 976.779 |
|  |  | rate | 0.0172 | 0.0028 | 0.0126 | 0.0236 |  |  |
|  | Log-logistic | shape | 2.5671 | 0.2098 | 2.1872 | 3.0131 | **929.809** | **936.365** |
|  |  | scale | 22.3274 | 1.2029 | 20.0899 | 24.8140 |  |  |
|  | Generalized gamma | mu | 3.2126 | 0.0809 | 3.0540 | 3.3711 | 938.649 | 948.483 |
|  |  | sigma | 0.6634 | 0.0645 | 0.5483 | 0.8027 |  |  |
|  |  | Q | 0.3593 | 0.2250 | -0.0816 | 0.8003 |  |  |

**Supplementary Table 3.** Associated Costs and Disutility of Grade ≥ 3 Treatment-Related Adverse Events

| **Adverse Event^a^** | **No. of patients (%)** | **Costs in 2021 USD^c^** | **Reference** | **Disutility^c^** | **Reference** |
| --- | --- | --- | --- | --- | --- |
| **Serplulimab plus chemotherapy^b^** | |  |  |  |  |
| Anaemia | 21 (5%) | 3,668 | (1) | 0.072 | (2) |
| Decreased neutrophil count | 33 (8%) | 3,184 | (1) | 0.348 | (3) |
| Decreased white blood cell count | 55 (14%) | 4,714 | (4) | 0.072 | (3) |
| Decreased platelet count | 24 (6%) | 6,928 | (5) | 0.108 | (6) |
| **Total** |  | 1,562 |  | 0.050 |  |
| **chemotherapy^b^** |  |  |  |  |  |
| Anaemia | 11 (6%) | 3,668 | (1) | 0.072 | (2) |
| Decreased neutrophil count | 17 (9%) | 3,184 | (1) | 0.348 | (3) |
| Decreased white blood cell count | 27 (14%) | 4,714 | (4) | 0.072 | (3) |
| Decreased platelet count | 16 (8%) | 6,928 | (5) | 0.108 | (6) |
| **Total** |  | 1,697 |  | 0.053 |  |

^a^Our analysis only included and evaluated grade ≥ 3 treatment-related adverse events.

^b^Number within treatment arm: serplulimab plus chemotherapy (N = 389), chemotherapy (N = 196).

^c^Calculated as an average cost of toxicity using the weighted frequency of occurrence. This value was used in the base-case model.

**References**

1. Wu B, Dong B, Xu Y, Zhang Q, Shen J, Chen H, et al. Economic Evaluation of First-Line Treatments for Metastatic Renal Cell Carcinoma: A Cost-Effectiveness Analysis in a Health Resource-Limited Setting. PLoS One (2012) 7(3):e32530. doi: 10.1371/journal.pone.0032530

2. Freeman K, Connock M, Cummins E, Gurung T, Taylor-Phillips S, Court R, et al. Fluorouracil Plasma Monitoring: Systematic Review and Economic Evaluation of the My5-Fu Assay for Guiding Dose Adjustment in Patients Receiving Fluorouracil Chemotherapy by Continuous Infusion. *Health Technol Assess* (2015) 19(91):1-321, v-vi. doi: 10.3310/hta19910

3. Nafees B, Lloyd AJ, Dewilde S, Rajan N, Lorenzo M. Health State Utilities in Non-Small Cell Lung Cancer: An International Study. Asia Pac J Clin Oncol (2017) 13(5):e195-e203. doi: 10.1111/ajco.12477

4. Wong W, Yim YM, Kim A, Cloutier M, Gauthier-Loiselle M, Gagnon-Sanschagrin P, et al. Assessment of Costs Associated with Adverse Events in Patients with Cancer. PLoS One (2018) 13(4):e0196007. doi: 10.1371/journal.pone.0196007

5. Zheng H, Xie L, Zhan M, Wen F, Xu T, Li Q. Cost-Effectiveness Analysis of the Addition of Bevacizumab to Chemotherapy as Induction and Maintenance Therapy for Metastatic Non-Squamous Non-Small-Cell Lung Cancer. Clin Transl Oncol (2018) 20(3):286-93. doi: 10.1007/s12094-017-1715-1

6. Konidaris G, Paul E, Kuznik A, Keeping S, Chen CI, Sasane M, et al. Assessing the Value of Cemiplimab for Adults with Advanced Cutaneous Squamous Cell Carcinoma: A Cost-Effectiveness Analysis. *Value Health* (2021) 24(3):377-87. doi: 10.1016/j.jval.2020.09.014
